# Far-Off Resonance: Multiwavelength Raman Spectroscopy Probing Amide Bands of Amyloid-β-(37–42) Peptide

**DOI:** 10.3390/molecules25153556

**Published:** 2020-08-04

**Authors:** Martynas Talaikis, Simona Strazdaitė, Mantas Žiaunys, Gediminas Niaura

**Affiliations:** 1 Department of Bioelectrochemistry and Biospectroscopy, Institute of Biochemistry, Life Sciences Center, Vilnius University, Saulėtekis Ave. 7, LT-10257 Vilnius, Lithuania; martynas.talaikis@gmc.vu.lt; 2 Department of Organic Chemistry, Center for Physical Sciences and Technology (FTMC), Saulėtekis Ave. 3, LT-10257 Vilnius, Lithuania; simona.strazdaite@ftmc.lt; 3 Institute of Biotechnology, Life Sciences Center, Vilnius University, Saulėtekis Ave. 7, LT-10257 Vilnius, Lithuania; mantas.ziaunys@gmc.vu.lt

**Keywords:** amyloid fibril, protein aggregation, secondary structure, β-sheet, amide II, Raman spectroscopy

## Abstract

Several neurodegenerative diseases, like Alzheimer’s and Parkinson’s are linked with protein aggregation into amyloid fibrils. Conformational changes of native protein into the β-sheet structure are associated with a significant change in the vibrational spectrum. This is especially true for amide bands which are inherently sensitive to the secondary structure of a protein. Raman amide bands are greatly intensified under resonance conditions, in the UV spectral range, allowing for the selective probing of the peptide backbone. In this work, we examine parallel β-sheet forming GGVVIA, the C-terminus segment of amyloid-β peptide, using UV–Vis, FTIR, and multiwavelength Raman spectroscopy. We find that amide bands are enhanced far from the expected UV range, i.e., at 442 nm. A reasonable two-fold relative intensity increase is observed for amide II mode (normalized according to the δCH_2_/δCH_3_ vibration) while comparing 442 and 633 nm excitations; an increase in relative intensity of other amide bands was also visible. The observed relative intensification of amide II, amide S, and amide III modes in the Raman spectrum recorded at 442 nm comparing with longer wavelength (633/785/830 nm) excited spectra allows unambiguous identification of amide bands in the complex Raman spectra of peptides and proteins containing the β-sheet structure.

## 1. Introduction

Protein misfolding and aggregation into insoluble amyloid fibrils is the central cause of many neurodegenerative diseases, including Alzheimer’s and Parkinson’s [1,2,3]. Despite the origin of the protein, all amyloid fibrils share a common cross-β-rich structure, stabilized by hydrogen bonds between polypeptide chains. Optical spectroscopy methods, particularly circular dichroism [4], infrared absorption (FTIR) [5,6], UV resonance Raman (UVRR) [7,8,9], non-resonance Raman [6,10], surface enhanced Raman [6,11], and vibrational sum-frequency generation spectroscopy [12] have proven to be successful in investigating the structural properties of peptides and proteins. Among these, UVRR spectroscopy is well established as a technique intrinsically sensitive to the secondary structure of the peptide backbone, because of selective resonance enhancement of amide bands. It also has the advantage of being able to probe aqueous samples without strong interference from the H_2_O signal, which is a common issue for FTIR. However, the application of the UVRR technique for routine analysis of peptides and proteins has been limited by the complexity and specifications of deep-UV lasers [13]. 

Raman spectroscopy was extensively applied for structural characterization [10,14,15], analysis of aggregation [14,16], and identification [17,18,19] of amyloid-β peptides. A recent critical review highlighted Raman spectroscopy’s ability to probe, by a label-free and nondestructive approach, the changes in secondary and tertiary structure at all stages of the fibrillation process [14]. The key vibrational indicators for the secondary structure are four amide bands: amide I (AI), amide II (AII), amide III (AIII), and amide S (AS) found in ca. 1610–1690, 1520−1564, 1220–1350, and 1374−1397 cm^−1^, respectively [20,21]. These bands are sensitive to secondary structure because (i) polypeptide’s geometry directly affects the force constants of the amide bond and (ii) each secondary structure element type participate in hydrogen bonding of different strength [20]. Alterations in force constants lead to differences in normal mode composition, which in turn is responsible for amide band frequency shift and intensity changes [21]. Thus, differences in geometry and hydrogen bonding strength are what make a reasonable change to the AI position (ca. 20 cm^−1^) comparing, for example, α-helix and β-sheet structures [20].

In general, AI and AIII are considered to be more structure-sensitive than AII. Therefore, numerous publications are mainly focused on analyzing AI and AIII as the main structure markers [13,20,21]. Nonetheless, some complementary data also could be obtained from AII. While the frequency of AII‘ mode (deuterated amide group, –CO–ND–) does not change significantly, its intensity is more strongly enhanced in β-sheet conformation and unordered structures [13,22]. Frushour et al. found that the AII band for β-sheet conformation is at least 50% stronger compared to that of α-helical polypeptides [22]. It is typically considered that the AII band is only detectable under resonance conditions [23,24]. Such an assumption is only partially true because in many cases AII is buried under other bands attributed to ring vibrations of the aromatic amino acids and deformations of NH_3_, making analysis of AII speculative or even leading to an erroneous assignment. The frequency of AII mode is primary sensitive to the H-bonding strength at N–H site of amide group [25]; higher frequency corresponds to stronger hydrogen bond formation. Thus, shifts of AII mode provides possibility to probe selectively the amide N–H hydrogen bonding. Stronger hydrogen bonds at the amide N–H site are responsible for higher observed AII frequency for β-sheet structure comparing with α-helix [9]. 

**Figure 1:**
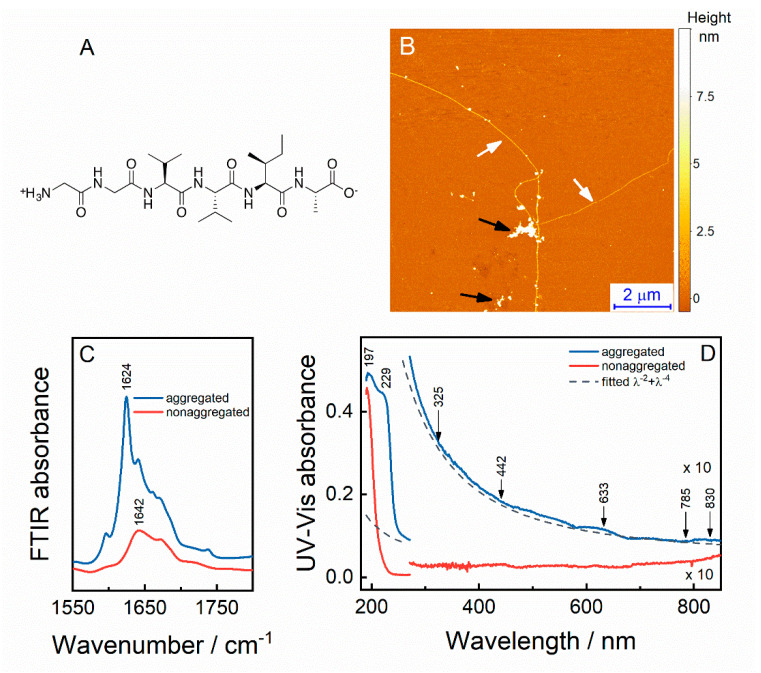
Molecular structure of amyloid-β-(37–42) motif, the GGVVIA (**A**). AFM image of aggregated GGVVIA peptide deposited on mica. Black arrows indicate peptide oligomers and white arrows indicate fibrils (**B**). FTIR spectra of aggregated (10 mg/mL) and non-aggregated (5 mg/mL) GGVVIA peptide in the amide I’ absorption region recorded in D_2_O solution (**C**). UV–Vis spectra of aggregated (10 mg/mL) and non-aggregated (0.01 mg/mL) GGVVIA peptide (**D**). Arrows indicate the wavelengths of laser radiation used in this study.

In this contribution, the glycyl-glycyl-valyl-valyl-isoleucyl-alanine peptide (GGVVIA or amyloid-β-(37–42); the structure is shown in Figure 1A) is studied using FTIR, UV–Vis, and multiwavelength Raman spectroscopy. The GGVVIA is a fragment of a full-length amyloid-β (Aβ) peptide and is located in the C-terminus segment of Aβ. It is known to aggregate into a fibrillar structure, consisting of parallel β-strands within the same β-sheet layer, while forming an anti-parallel orientation between adjacent β-sheet layers [26]. The structure is also supported by the hydrophobic steric-zipper formed by V3, V4, and I5 amino acids of neighboring peptide sheets [27]. It is believed that the GGVVIA segment is central in the fibrillation process of a full-length Aβ since it has the highest propensity to aggregate [27,28]. Here, we monitor the state of GGVVIA aggregation by using structure-sensitive Raman spectroscopy. We show that Raman amide bands of the GGVVIA are excitation-wavelength sensitive in a spectral range beyond UV. This finding is especially true for the AII band, which shows the highest intensity enhancement when comparing spectra excited with blue (442 nm) and red (633, 785, and 830 nm) laser radiations. Such a wavelength sensitivity may be taken advantage of in situations where a direct assignment of AII of Aβ and other polypeptides and proteins is not straightforward.

## 2. Results

FTIR spectra were recorded to find the concentrations at which the GGVVIA peptide is in aggregated and non-aggregated states (Figure 1C). We found that the sample with a concentration of 10 mg/mL has a sharp absorption peak at 1624 cm^−1^, which is a clear indication of an aggregated β-sheet structure [29]. Once the concentration is halved, the peak drops in intensity, broadens and upshifts to 1642 cm^−1^, signaling the dissociation of its secondary structure (unordered structure). Therefore, we assign the concentration of 5 mg/mL and below to the non-aggregated state of the peptide.

**Figure 2:**
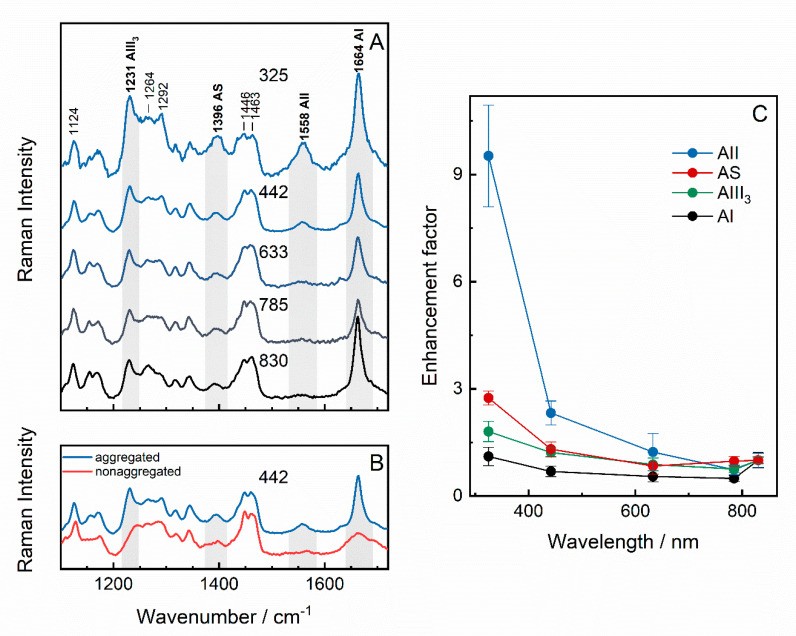
Raman spectra of aggregated (20 gm/mL) GGVVIA peptide in H_2_O excited at 325, 442, 633, 785, and 830 nm (**A**) and Raman spectra of aggregated and non-aggregated (5 mg/mL) GGVVIA in H_2_O excited at 442 nm. Spectra are normalized with respect to the peak intensity at 1463 cm^−1^; the spectral intensity is retained in both (**A**) and (**B**) panels. The (**C**) panel shows the dependence of the enhancement factor of AII, AS, AI, and AIII_3_ bands on the excitation wavelengths.

Figure 1D shows UV–Vis absorption spectra of aggregated (10 mg/mL) and non-aggregated (0.01 mg/mL) GGVVIA peptide. During the acquisition of the aggregated sample, we observed light scattering from the suspension, visible as a steadily increasing absorption towards shorter wavelengths. We suspect scattering from both the small particles of peptide aggregates (Rayleigh scattering; RS) and the larger oligomer tapes (Tyndall scattering; TS). The distinct nature of these phenomena results in different scattering efficiency with RS following the *λ*^−4^ and TS following the *λ*^−2^ trend [30]. Therefore, the scattering becomes increasingly intensified towards the blue portion of the spectrum. The AFM image in Figure 1B of aggregated GGVVIA sample deposited on mica indicates long fibrils with length in micrometer scale, alongside with clusters of smaller aggregated species. Based on the AFM data, we assume the RS and TS for the aggregated sample. Therefore, we fitted and subtracted the sum of RS and TS contours, *λ*^−2^ + *λ*^−4^, from the absorption spectrum of the aggregated peptide (Figure 2D, blue line), which flattened the curve in the visible range. No scattering was observed for the non-aggregated peptide solution due to a much lower peptide concentration and absence of aggregates.

Typically, an electronic excitation of the peptide backbone occurs in the range of 130–230 nm [31]. Here, we show that absorption bands emerge at 197 and 229 nm for aggregated and at 195 nm for non-aggregated peptide. Expectedly, no absorption is detected in the rest of the spectra since the GGVVIA is composed of aliphatic amino acids and glycine, which do not possess electronic transitions in the visible spectral range.

We further examine the solutions of aggregated and non-aggregated peptide (5 and 20 mg/mL) by using multiwavelength Raman spectroscopy excited with 325, 442, 633, 785, and 830 nm laser wavelengths. According to the UV–Vis data, the resonance Raman scattering should not take effect at any of these wavelengths. Figure 2A shows the Raman spectra of GGVVIA aggregates excited between 325 and 830 nm. The spectra are normalized according to the δCH_2_/δCH_3_ vibration at 1463 cm^−1^. Assignments of the spectral bands are based on previous publications [9,13,20,32,33,34,35,36,37] and are given in Table 1. Since GGVVIA consists of simple aliphatic amino acids, the spectrum of the peptide appears to be fairly clear. The sharp AI band located at 1664 cm^−1^ is primarily due to C = O stretching. AII mode at 1558 cm^−1^ derives from the vibrational coupling of out-of-phase C–N stretching and in-plane N–H bending motions. Coupling with N–H deformation vibration is confirmed by analysis of Raman spectra of aggregated and non-aggregated peptides in D_2_O solution (Figure S1, Supplementary Materials); well-defined AII mode at 1558 cm^−1^ completely disappears in the spectrum recorded in D_2_O solvent. The new AII’ mode is difficult to assign, because of strong CH_2_/CH_3_ deformation bands in the expected spectral interval (near 1450−1480 cm^−1^). The strong negative feature in the difference (H_2_O minus D_2_O, see Supplementary Materials Figure S1) spectra might be associated with HOD bending vibration of solvent. The AII’ mode is expected to appear at lower wavenumbers because of uncoupling with N–H deformation motion [9]. This mode contains high C–N motion contribution and usually appears as a strong band in deep-UV resonance Raman (DUVRR) spectra [9]. AIII mode is the most complex band and contains contributions from AIII_3_, AIII_2_, and AIII_1_ modes centered at 1231, 1264, and 1292 cm^−1^ [33,35]. AIII originates from in-phase C–N stretching vibration coupled with N–H in-plane bending. The 1396 cm^−1^ band is assigned to the symmetric bending of C_α_–H, named amide S (AS) [33,35]. Contributions from methyl, methylene, and C–H deformations of valine, isoleucine, and alanine are visible at 1435, 1446, and 1463 cm^−1^. 

Aggregated and non-aggregated peptide show differences in all amide regions of the 442-nm-excited Raman spectra (Figure 2B). The AI band decreases in intensity and its width determined as full width at half maximum (FWHM) increases from 16 cm^−1^ for aggregated to 50 cm^−1^ for non-aggregated species. Next, intensity of the AII mode drops substantially as the peptide disaggregates. Finally, AIII_3_ loses intensity, its FWHM increases, and the wavenumbers shift to higher energy. Importantly, formation of β-sheet secondary structure results in considerable enhancement of AII band comparing with unordered structure; augmentation by 3.9 and 2.8 times was obtained for 442- and 785-nm-excited spectra, respectively. 

Comparing spectra collected with different laser excitation wavelengths, we observed an interesting phenomenon. Although excitation happens far from electronic absorption, an intensification of amide bands appears when excitation wavelengths change from 633 to 442 and finally to 325 nm.

The dependence of the normalized intensity of AI, AII, AIII_3_, and AS bands on excitation wavelengths is shown in Figure 2C. Intensity normalization was done with respect to the methyl/methylene deformation band at 1463 cm^−1^. The mean value ± SD is calculated from four measurements for 325 nm excitation and three measurements for 442–830 nm excitations. All the selected bands show similar trend of intensity augmentation towards the UV portion of the spectrum. To further confirm observed phenomenon, we conducted a similar analysis by using two other internal vibrational standards: (a) 1124 cm^−1^ band related with rocking r(CH_3_) + δ(CCH) vibration of side chains (Table 1), which is not sensitive to the aggregation of the peptide (Figure 2B) and appears in uncrowded spectral region, and (b) deformation vibrational band of water, δ(HOH), near 1641 cm^−1^ (Figure S2). Excitation wavelength-dependent changes in amide vibrational bands obtained by using these two internal standards were found to be very similar (Figure S3) to the trend displayed in Figure 2C, thus confirming the consistency of observed phenomenon. 

## 3. Discussion

In this work, two factors were found to be responsible for considerable enhancement of AII mode in Raman spectra excited in the visible spectral region: (i) aggregation of disordered peptides into the β-sheet secondary structure, and (ii) shift of laser excitation line to shorter wavelengths. 

FTIR data presented in Figure 1C show that at a concentration of 10 mg/mL, the GGVVIA peptide is aggregated and at 5 mg/mL (and below), the peptide becomes disaggregated and possesses unordered structure. Based on these findings, we continued with UV–Vis absorption measurements of GGVVIA solutions. The UV–Vis absorption spectra show peaks centered at 229 and 197 nm for aggregated peptide and at ca. 195 nm for non-aggregated (Figure 1D). These peaks in simple amides are linked with n→π* (for 229 nm) and π→π* (for 195–197 nm) electronic transitions of the peptide bond [31]. The n→π* transition is characteristic for protein aggregates; it derives from the overlap between the lone electron pair orbital (n) of the carbonyl oxygen and the antibonding orbital (π*) from another carbonyl group [38,39]. Although appearing mostly for α-helices, the transition with less extent is also observed for β-sheet structures [38].

In our case the peptide exhibits no absorption beyond 250 nm since it lacks any chromophore units or aromatic amino acids. This implies that visible range lasers can excite only non-resonance Raman scattering from the sample. Therefore, resonance Raman studies on peptide backbone could have been accomplished only in UV spectral regime [9,13,20,24,31,32,35,40]. Indeed, strongly enhanced (10^2^–10^6^ times) peptide backbone modes were observed at deep-UV excitation wavelengths (below 220 nm) corresponding to π_2_→π*_3_ dipole-allowed electronic transition with maximum at 188 nm [21]. All vibrational modes containing high contribution of peptide bond C–N stretching motion are resonantly enhanced as molecular geometry changes along the C–N bond during the electronic excitation [41]. 

To our surprise, far from resonance conditions (at 325 and 442 nm), appreciable Raman intensity enhancement is observed for amide bands (Figure 2A). We note that enhancement effect is not visible for non-aggregated peptide; all amide bands appear weak in Raman spectra excited at 442 nm (Figure 2B). The more quantitative dependence of the normalized intensity of amide bands on excitation wavelengths for aggregated peptides are given in Figure 2C. The excitation profiles of amide bands seem to adopt exponential dependence towards the blue portion of spectra and somewhat flattened out in the range over 633 nm. A moderate augment of peak intensities for AI, AS, and AIII_3_ modes by factors of 1.1, 2.8, and 1.8, respectively, is obtained comparing the spectra collected at 325 nm with the ones at 830 nm. Yet, even stronger effects is observed for AII, where an increase by a factor of 9.5 (±1.4) was found for the transition from 830 to 325 nm excitation. Quite a reasonable intensification of 2.3 (±0.3) was also observed for the spectra collected at 442 nm comparing with 830 nm, and 1.9 (±0.3) for 633 to 442 nm transition. 

Previously, Asher and coworkers have shown that AII vibration is the most strongly enhanced in comparison with other amide bands for the model compound *N*-methylacetamide, α-helical, and polyproline II-like peptide conformations under resonance conditions [31,40]. The AII enhancement was attributed to the displacement of the C–N bond, which is the main contributor to the AII vibration, along the transition from the electronic ground to the excited state. The excitation induced displacement results in increased UVRR cross-section [42]. This is not the case for the AI, where dominant C=O stretching has a decreased displacement along the bond during the transition. Here, we would like to stress that the enhancement effect for AII of GGVVIA peptide is visible far outside the UV range, at 442 nm. Recently, the Raman AII band for β-sheet-rich α-synuclein was observed at 514 nm. [43]. The authors have hypothesized, that the band emerges due to vibrational coupling introduced by secondary structure alignment, and not due to the pre-resonance effect. Almost two-fold increase found in our study while going from 633 to 442 nm excitation is rather significant, thus we believe it could be beneficial in identifying the AII band where such an assignment is not straightforward. Multiwavelength Raman spectroscopy analysis of aggregated and non-aggregated peptides holds some similarities as well as differences comparing with DUVRR studies of peptides. Similarly, as in the case of DUVRR spectra, the amide vibrational modes containing substantial C–N stretching contribution are preferentially enhanced; especially this holds for AII mode containing high C–N motion. However, contrary to DUVRR studies, Raman spectra excited with visible radiation show enhancement only in the case of peptides aggregated into the β-sheet structure, while strong amide bands were detected in deep-UV-excited resonance Raman spectra for unordered secondary structures [9]. 

The origin of underlying phenomena currently is not clear. It might be related to the quantum confinement effect present in peptide aggregates with dimensions of several nanometers [44]. The charge delocalization phenomena in strongly hydrogen bonded molecular structures should also be considered [45]. Alternatively, the hidden electronic transition for aggregated amyloid–β peptides might be related to the observed phenomenon. Such electronic transition is difficult to observe by UV–Vis measurements, because of the complications of light scattering in the presence of aggregated peptides. Recently, intrinsic blue-green fluorescence was observed for peptides and proteins aggregated into the amyloid fibrils irrespectively on the presence of aromatic amino acid residues [46,47,48,49]. The phenomenon was found to be strictly connected with the formation of a β-sheet secondary structure. It was suggested that electron delocalization, resulting from the formation of strong hydrogen bonds in the β-sheet structure, is responsible for the appearance of low energy electronic transition [46]. Indeed, such phenomenon might be important in excitation wavelength-dependent enhancement of amide modes observed in this work. However, the molecular origin of blue-green fluorescence and related electronic transition in amyloid structures currently is far from complete understanding and is under excessive development [48,49]. 

Summarizing, we observe the peculiar response of Raman amide bands, especially AII, to the wavelengths of the excitation laser in the visible range for β-sheet aggregates of the GGVVIA peptide. Considerable enhancement of the AII band was observed in the Raman spectra excited in the visible spectral region due to aggregation of disordered peptides into the β-sheet secondary structure. Deep-UV resonance Raman studies have evidenced the highest intensity enhancement for the β-sheet and unordered secondary structures [9,32]. In contrast, our study evidences relative enhancement in amide bands only for the β-sheet secondary structure. We believe that these findings may be useful in facilitating the assignment of amide bands and especially AII mode of peptides and small proteins with β-sheet structure.

## 4. Materials and Methods

### 4.1. Materials

The synthetic GGVVIA peptide was purchased from CASLO (Denmark) and used without any further purification (≥98.1%, as lyophilized chloride salt). Ultra-pure water (18.2 MΩ cm) from the Direct-Q 3UV (Merck, Darmstadt, Germany) water purification system was used for measurements. D_2_O (≥99.9%) was purchased from Sigma-Aldrich (Oakville, Canada).

### 4.2. UV–Vis Spectroscopy

Electronic absorption spectra were recorded of aggregated GGVVIA peptide (10 mg/mL; 19.4 mM) in quartz cell with an optical path length of 0.1 cm and non-aggregated peptide (0.01 mg/mL; 0.02 mM) in quartz cell with an optical path length of 1 cm. In both cases, reference cell was filled with H_2_O. Spectra were collected using PerkinElmer Lambda 25 (Buckinghamshire, UK) in a spectral range from 900 to 190 nm, with a scanning rate of 240 nm/s.

### 4.3. FTIR Spectroscopy

FTIR spectra were taken with Bruker Vertex 80v spectrometer (Ettlingen, Germany) with liquid nitrogen cooled MCT detector. To reduce water vapor absorption, the spectrometer and sample compartment were vacuumed. Solutions containing 5 and 10 mg/mL (9.7 and 19.4 mM) of peptide in D_2_O were recorded in 100 μL CaF_2_ transmission cell. The CaF_2_ window was used as a reference. Each spectrum was collected using 128 scans at 1 cm^−1^ resolution. We subtracted D_2_O from the peptide spectra.

### 4.4. Atomic Force Microscopy

The solution of the aggregated peptide (10 mg/mL in D_2_O) was incubated at room temperature for three days. Then the solution was diluted 10 times with D_2_O and deposited onto a freshly cleaved mica surface and incubated for 1 min. Finally, the sample was rinsed with 1 mL of ultra-pure water and dried under gentle airflow. The AFM images were taken with a Dimension Icon (Bruker, Karlsruhe, Germany) atomic force microscope, which operated in tapping mode and was equipped with a silicon cantilever Tap300AI-G (40 N m^−1^, Budget Sensors, Sofia, Bulgaria) with a tip radius of curvature of 8 nm. High-resolution (1024 × 1024 pixels) images were collected at 0.5 Hz scan rate. AFM images were flattened using SPIP (Image Metrology, Lyngby, Denmark) software.

### 4.5. Raman Spectroscopy

Multiwavelength Renishaw InVia spectrometer (Wotton-under Edge, UK) was used in the study. For Raman measurements, solutions of non-aggregated (5 mg/mL, 9.7 mM) and aggregated (20 mg/mL, 38.8 mM) peptide in H_2_O were prepared and placed into a quartz cell. The parameters used at each laser excitation wavelength are given in Table 2. We present combined Raman spectra from three or four independent measurements, each of which took 1 h (360 spectra of 10-s integration time were coadded), collected at the different location of the same sample. No temporal spectral changes were detected during the accumulation of the Raman spectra; the first and the last spectra were identical, indicating the preservation of the sample integrity.

### 4.6. Spectra Manipulation

Raman spectra contained a high contribution from H_2_O; therefore, the pure H_2_O spectrum was subtracted in a post-experiment treatment. Next, the spectra were intensity-corrected by using a light source (Raman Calibration Kit, SN WL043, Tornado, Toronto, Canada) because of an uneven throughput efficiency of CCD detector over the whole spectral range. Raman wavenumbers were calibrated according to the polystyrene spectrum, followed by subtraction of polynomial function baseline. Spectra were normalized according to the intensity of δ(CH_2_/CH_3_) band at 1463 cm^−1^. Statistical data were presented as an average of three-four measurements ± standard deviation.

## Figures and Tables

**Table 1:** Assignments of Raman spectral bands of aggregated GGVVIA peptide recorded at 325 nm excitation.

Wavenumber, cm^−1^	Assignment	References
1124	r(CH_3_), δ(CCH)	[37]
1231	AIII_3_; β-sheet	[9,20,33,34]
1264	AIII_2_	[33]
1292	AIII_1_	[33]
1320	tCH_2_, wCH_2_	[33]
1346	δCH_2_	[36]
1396	AS	[9,20,35]
1435	δCH_2_	[36]
1446	δCH_2_	[36]
1463	δCH_2_, δCH_3_	[36]
1558	AII	[9,13,20,32]
1664	AI; β-sheet	[13,20,34]

Abbreviations: r, rocking; t, twisting; w, wagging; δ, deformation.

**Table 2:** Setup of the Raman system.

Laser Line, nm	Power at Sample, mW	Grating, Lines/mm	Objective, Mag. (NA); Spectral Range
325	4.3	3600	Thor Labs, 15× (0.32); UV
442	53	2400	Leica, 5×, (0.12); Vis
633	9.4	1200	Leica, 5×, (0.12); Vis
785	92	1200	Olympus, 50× (0.65); IR
830	166	830	Olympus, 50× (0.65); IR

## Supplementary Materials

The following are available online. Figure S1: Raman spectra of non-aggregated and aggregated GGVVIA peptides in H_2_O and D_2_O as well as the difference spectra of H_2_O minus D_2_O; Description of Raman enhancement factor analysis by using protein band at 1124 cm^−1^ and water deformation vibration band at 1641 cm^−1^; Figure S2: Construction of the difference spectrum by subtracting the water spectrum from the spectrum of peptide in water, with indicated reference bands. The difference spectrum is fitted by Gaussian-Lorentzian shape components; Figure S3: Wavelength-dependent enhancement factors for selected vibrational modes. The relative intensities for spectral bands were acquired with respect to the spectral bands near 1124 cm^−1^ and the deformation vibration of water near 1641 cm^−1^.
