# Supplementary material for: Far-Off Resonance: Multiwavelength Raman Spectroscopy Probing Amide Bands of Amyloid-β-(37–42) Peptide
Source: Molecules. 2020 Aug 4;25(15):3556. doi: 10.3390/molecules25153556 (PMC7435454; doi:10.3390/molecules25153556)
Supplement: Supplementary file 1 [file molecules-25-03556-s001.pdf]

Supplementary Material

Far-off Resonance: Multiwavelength Raman Spectroscopy Probing Amide bands of Amyloid-β-(37-42) Peptide

Martynas Talaikis[1], Simona Strazdaitė[2], Mantas Žiaunys[3], and Gediminas Niaura[2,*]

1 Department of Bioelectrochemistry and Biospectroscopy, Institute of Biochemistry, Life Sciences Center, Vilnius University, Saulėtekis Ave. 7, LT-10257 Vilnius, Lithuania; martynas.talaikis@gmc.vu.lt
2 Department of Organic Chemistry, Center for Physical Sciences and Technology, Saulėtekis Ave. 3, LT-10257, Vilnius, Lithuania; simona.strazdaite@ftmc.lt
3 Institute of Biotechnology, Life Sciences Center, Vilnius University, Saulėtekis Ave. 7, LT-10257 Vilnius, Lithuania; mantas.ziaunys@gmc.vu.lt
* Correspondence: gediminas.niaura@ftmc.lt

RESULTS

H/D exchange.

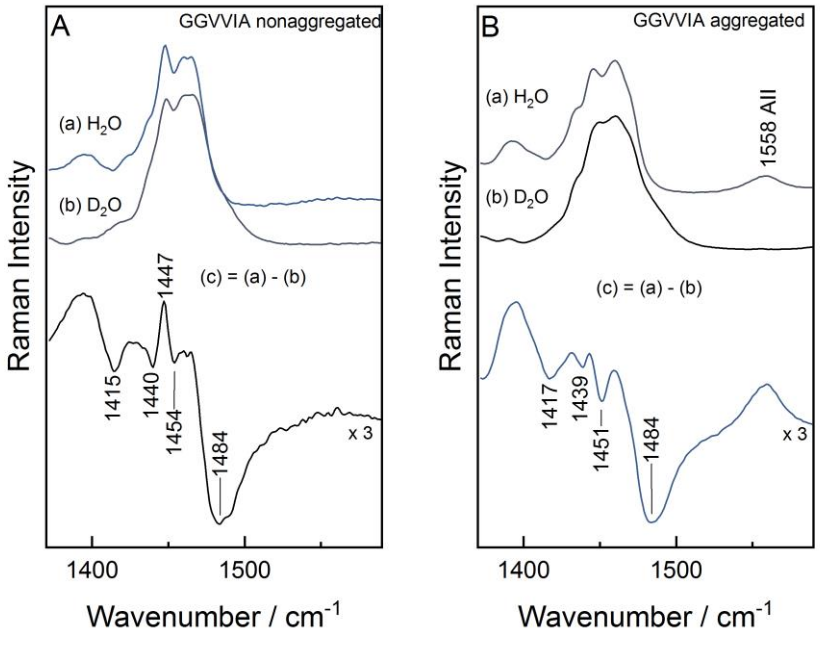

Figure S1. Raman spectra of nonaggregated (A), and aggregated (B) GGVVIA peptides in H_2O (a), and D_2O (b), as well as the difference spectra of H_2O minus D_2O (c). The spectra were collected using HyperFlux PRO Plus Raman spectrometer at 785 nm excitation.

Raman Enhancement factor analysis by using the protein band at 1124 cm^{-1} and water deformation vibration band at 1641 cm^{-1}.

To avoid erroneous interpretation of the experimental results, we calculate the enhancement factors with respect to two alternative intensity standards: the side-chains' r(CH_3) and δ(CCH) vibration near 1124 cm^{-1} [S1] and δ(HOH) band near 1641cm^{-1} (Figure S2). We chose the band near 1124 cm^{-1} because it is aggregation and excitation wavelength independent, and is located in a rather unoccupied spectral region. The enhancement factors calculated in Figure S3 compare well with the ones presented in Figure 2C.

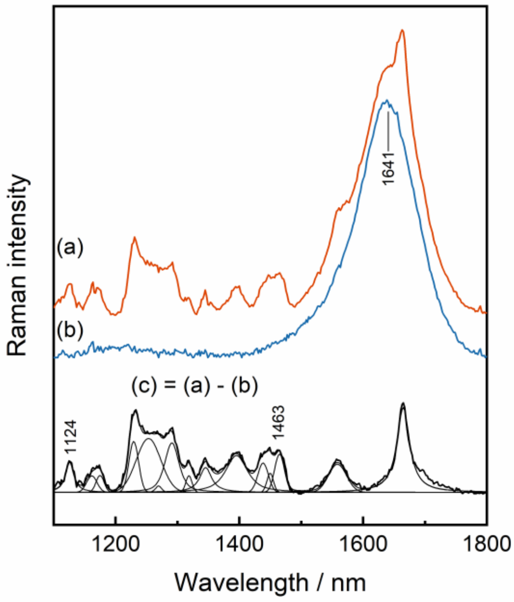

Figure S2. Construction of the difference spectrum (c) by subtracting the water spectrum (b) from the spectrum of the peptide in water (a), with indicated reference bands. The difference spectrum is fitted with Gaussian-Lorentzian shape components. The excitation wavelength is 325 nm.

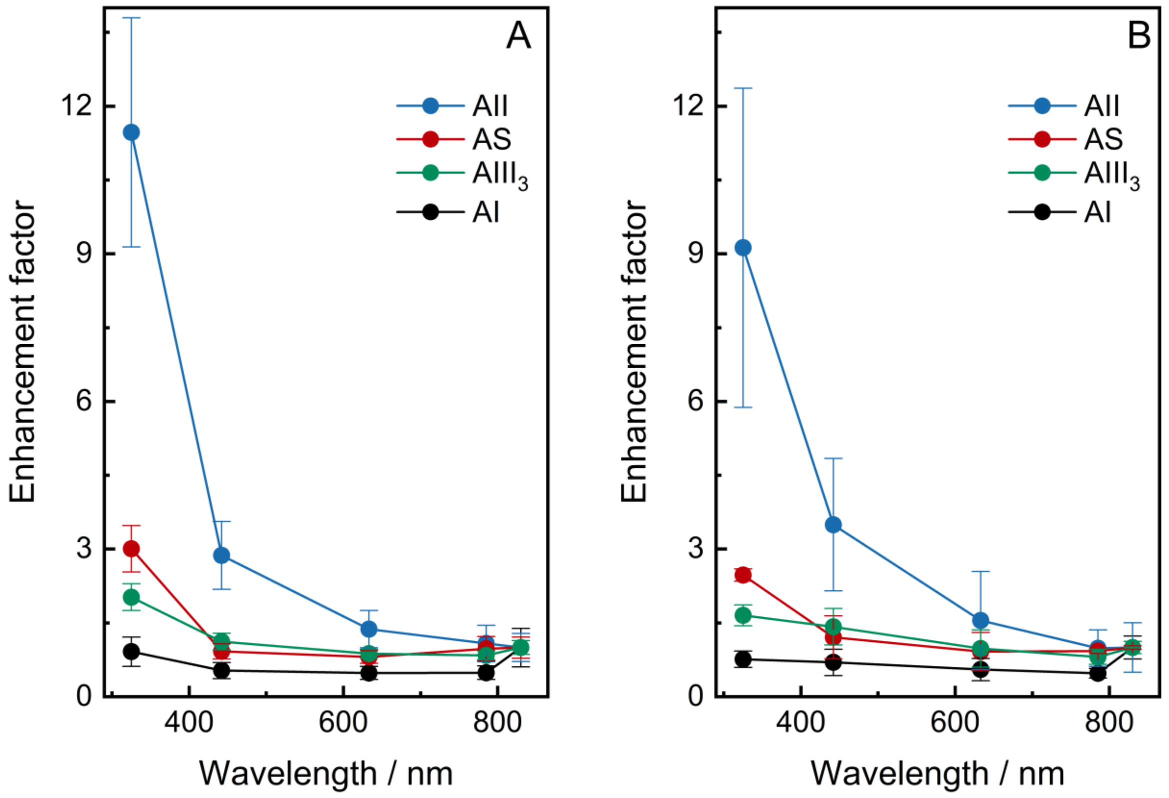

Figure S3. Wavelength-dependent enhancement factors for selected vibrational modes. The relative intensities for spectral bands were acquired with respect to the spectral band near 1124 cm^{-1} (A) and the deformation vibration of water near 1641 cm^{-1} (B).

METHODS

H/D exchange.

The H/D exchange in aggregated and nonaggregated GGVVIA peptide samples was monitored using HyperFlux PRO Plus (Tornado Spectral Systems, Canada) Raman spectrometer at 785 nm excitation, with laser power of 495 mW, which was transmitted via optical cable. The acquisition time was 1 hour.

Enhancement factor calculation.

The enhancement factors were calculated by comparing the relative intensity of a particular spectral mode at a given wavelength with the one obtained at 830 nm. For accuracy, we chose three reference bands to calculate the relative intensity (Figure S2). The reference bands at 1124 and 1463 cm^{-1} belong to peptide vibrations, and the band at 1641 cm^{-1} is assigned to the deformation of water molecules. The enhancement factor dependencies on the wavelength are presented in Figure 2C and Figure S3.

REFERENCES

S1. Hernandez, B., Pflüger, F., Nsangou, M. & Ghomi, M. Vibrational analysis of amino acids and short peptides in hydrated media. IV. Amino Acids with Hydrophobic Side Chains: L-Alanine, L-Valine, and L-Isoleucine. *J. Phys. Chem. B* **2009**, *113*, 3169–3178.
